# Expression of TNRC6 (GW182) Proteins Is Not Necessary for Gene Silencing by Fully Complementary RNA Duplexes

**DOI:** 10.1089/nat.2019.0815

**Published:** 2019-12-02

**Authors:** Zhongtian Liu, Samantha T. Johnson, Zhiying Zhang, David R. Corey

**Affiliations:** ^1^ College of Animal Science and Technology, Northwest A&F University, Shaanxi, China.; ^2^ Departments of Pharmacology and Biochemistry, UT Southwestern Medical Center at Dallas, Dallas, Texas.

**Keywords:** trinucleotide repeat containing 6, RNA interference, argonaute, nuclear/cytoplasm localization

## Abstract

The trinucleotide repeat containing 6 (TNRC6) family of proteins are core components of RNA interference (RNAi) and consist of three paralogs (TNRC6A, TNRC6B, and TNRC6C). The TNRC6 paralogs associate with argonaute (AGO) protein, the core RNAi factor, and bridge its interactions with other proteins. We obtained TNRC6A and TNRC6B single and double knockout cell lines to investigate how the TNRC6 paralogs contribute to RNAi. We found that TNRC6 proteins are not required for gene silencing when duplex RNAs are fully complementary. TNRC6 expression was necessary for regulation by a microRNA. TNRC6A, but not TNRC6B, expression was necessary for transcriptional activation by a duplex RNA targeting a gene promoter. By contrast, AGO2 is required for all three gene expression pathways. TNRC6A can affect the Dicer localization in cytoplasm versus the nucleus, but none of the three TNRC6 paralogs was necessary for nuclear localization of AGO2. Our data suggest that the roles of the TNRC6 paralogs differ in some details and that TNRC6 is not required for clinical therapeutic silencing mechanisms that involve fully complementary duplex RNAs.

## Introduction

Human GW182 protein, also known as trinucleotide repeat containing 6 protein A (TNRC6A), plays an important role in RNA interference (RNAi) [1–3]. TNRC6A and its paralogs TNRC6B and TNRC6C are multidomain proteins that act as scaffolds to organize supramolecular complexes [4–10]. The N-terminal repeat GW-repeat containing region of the TNRC6 proteins binds to argonaute (AGO) protein [9,11–14]. The complex between AGO and guide RNA is responsible for sequence-selective recognition, whereas the TNRC6 paralogs recruit interacting factors such as the CCR4-NOT complex to mediate gene expression [15–18].

Each TNRC6 paralog includes an AGO binding domain, a silencing domain containing a PABPC1 interacting motif and an RNA recognition motif, an ubiquitin-associated domain, and a glutamine-rich region [19]. The paralog's amino acid sequence identity is 19.5%, with 418 identical positions and 413 similar positions. Each pairing of the paralogs shows ∼40% sequence identity. For comparison, the four AGO proteins have a 69.5% identity.

The involvement of the TNRC6 protein family in microRNA (miRNA) function is well known but the individual roles of TNRC6A, TNRC6B, and TNRC6C in RNAi are not [3,18,20]. Previous studies have suggested that the TRNC6 paralogs play critical roles in controlling gene translation and gene transcription [3,21,22]. These studies, however, used small interfering RNAs (siRNAs) to knockdown TNRC6A, TNRC6B, and TNRC6C. Substantial residual *TNRC6* gene expression remained, clouding the ability to draw definitive conclusions regarding individual contributions of the paralogs.

**FIG. 1.**
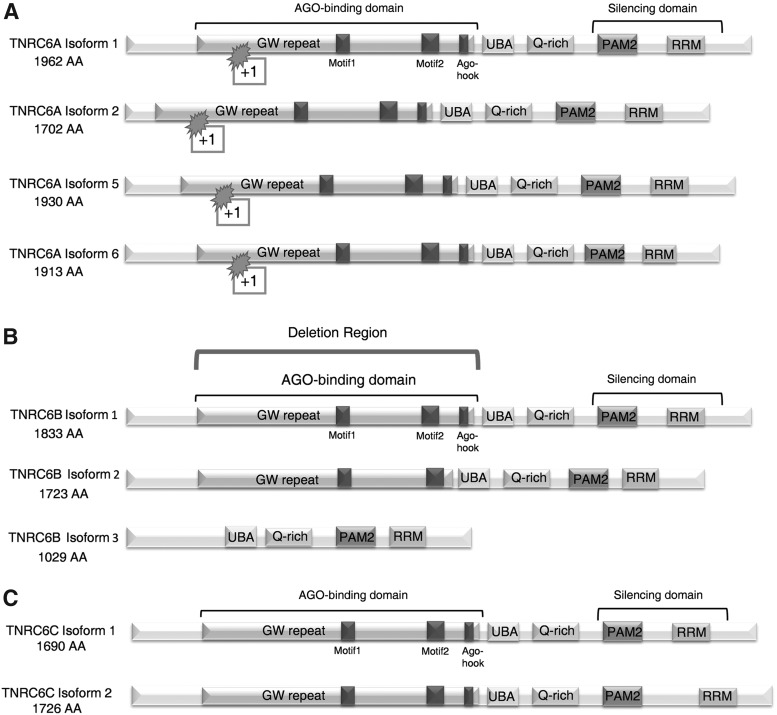
Diagrams of TNRC6 protein paralogs' domains and mutations. **(A)** The major isoforms of TNRC6A (isoforms 1, 2, 5, and 6) have been mutated by insertion of 1 base pair into the AGO binding domain region. **(B)** The major isoforms of TNRC6B have a large 95,481 base-pair deletion of the AGO binding domain region. TNRC6B isoform 3 does not contain the AGO binding region and is not affected by this deletion. **(C)** Two isoforms of TNRC6C. AGO, argonaute; TNRC6, trinucleotide repeat containing 6.

**FIG. 2.**
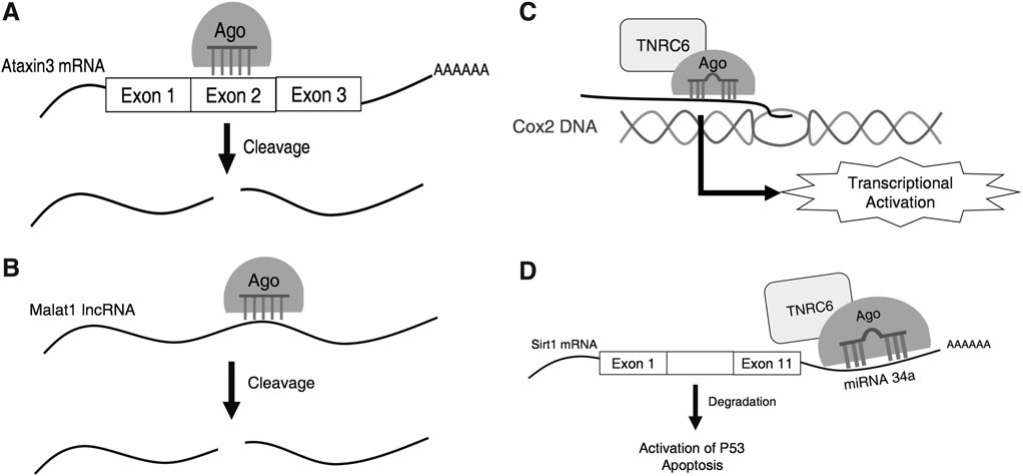
Diagram of the small RNA systems used to evaluate TNRC6 involvement in RNAi processes. **(A)** AGO2 loaded with siATX-3 targets and cleaves ATX-3 mRNA causing siRNA knockdown of ATX-3 in the cytoplasm. **(B)** AGO2 loaded with siMalat1 targets and cleaves Malat1 ncRNA causing siRNA knockdown of Malat1 in the nucleus. **(C)** AGO2 loaded with a small RNA binds to a sense transcript that overlaps the *COX-2* promoter. This causes further activation of gene transcription. **(D)** AGO2 loaded with miR34a targets and causes the degradation of Sirt1 mRNA. This causes the activation of P53 and apoptosis. ATX-3, ataxin-3; COX-2, cyclooxygenase-2; mRNA, messenger RNA; miRNA, microRNA; ncRNA, noncoding RNA; RNAi, RNA interference; siRNA, small interfering RNA.

To obtain more definite insights into the roles of the TNRC6 paralogs, we obtained CRISPR knockout cell lines lacking TNRC6A, TNRC6B, and both TNRC6A and TNRC6B (Fig. 1). Using these knockout cell lines, we have studied the individual functions of TNRC6A and TNRC6B during silencing by siRNA in the cytoplasm and nucleus, transcriptional silencing by small RNAs, and translational silencing by miRNA (Fig. 2). We find that TNRC6 protein is not needed for therapeutic gene silencing by fully complementary duplex RNAs.

## Materials and Methods

### Double-stranded RNAs and primers

RNA oligonucleotides and primers were purchased from Integrated DNA Technologies (Coralville, IA; Supplementary Tables S1 and S2). Double-stranded RNAs were prepared by mixing the two RNA strands and annealing them in 2.5 × phosphate buffer solution (PBS). Stock solutions (20 μM) were prepared for transfection in cell culture.

### Cell culture and transfection

Wild-type HCT116 cells were obtained from Horizon and originated from the American Type Tissue Culture Collection. These parental HCT116 cells were used to knock out the *TNRC6A*, *TNRC6B*, and *TNRC6A* and *TNRCB* genes and the knockout lines were obtained from GenScript (Supplementary Fig. S1; Supplementary Table S3). The AGO2 knockout HCT116 cells were a gift from the laboratory of Dr. Joshua Mendell [23]. HCT116 wild-type and knockout cells were cultured in McCoy's 5A medium (Sigma-Aldrich) supplemented with 10% fetal bovine serum (Sigma-Aldrich) in 37°C 5% CO_2_.

Lipofectamine RNAi MAX (Invitrogen) was used for all transfections of duplex RNAs. For forward transfections, cells were plated into six-well plates (Costar) 24 h before transfection. Wild-type, TNRC6A^−/−^, TNRC6B^−/−^, and AGO2^−/−^ cells were seeded at 150,000 cells per well and TNRC6AB^−/−^ cells were seeded at 250,000 cells per well. More cells were needed for TNRC6AB^−/−^ culture because the cells grow slowly (Supplementary Fig. S2). For transfection of duplex RNAs, lipid was added into OPTI-MEM (Invitrogen) with duplex RNA then added to a final volume of 1.25 mL. For all transfections, duplex RNA was added to a final concentration of 20 nM. For reverse transfection, wild-type, TNRC6A^−/−^, TNRC6B^−/−^, and AGO2^−/−^ cells were seeded at 150,000 cells per well and TNRC6AB^−/−^ cells were seeded at 175,000 cells per well into 6-well plates in 1 mL culture media. At the same time, lipofectamine RNAi MAX (Invitrogen) and duplex RNA were added into OPTI-MEM in a final volume of 1 mL and then added into cells as total volume of 2 mL. For double-transfection experiments, the first transfection was forward transfection, 2 days later the second reverse transfection was carried out. Medium was changed 24 h after transfection and every 2 days. For measuring the MALAT-1 gene RNA level expression, the cells were harvested 2 days after transfection, and cells were harvested 3 days after transfection for the other RNA analysis by quantitative PCR (qPCR) and 4 days after transfection for protein analysis by western blot.

### miR-34a assay

Cells were forward transfected as already described. Three days after transfection, we harvested the medium, the PBS wash, and the trypsinized cells to collect all cells, including dead cells, for counting, to and retain any floating dead cells. Cells were collected together, spun at 500*g* for 5 min, supernatant was removed, and resuspended in 1 mL of cell culture medium. Cells were mixed together with equal volume of trypan blue (Sigma) and were counted using cell counter (TC20™ Automated Cell Counter; Bio-Rad). The percentage of live cells was calculated as a relative value to the control sample.

### qPCR analysis

Total RNA was extracted using TRIzol (Life Technologies) and 2 μg of RNA was subjected to DNase I (Worthington Biochemical Corp) treatment. Complementary DNA (cDNA) was prepared using the High Capacity cDNA Reverse Transcription Kit (Life Technologies). After dilution of the cDNA sample, qPCR was performed on a CFX96 real-time PCR system (Bio-Rad) using iTaq SYBR Green Supermix (Bio-Rad). Data were normalized relative to levels of HPRT, SNRN, and RPL19 messenger RNA (mRNA). The qPCR cycles are as follows: 50°C for 2 min, 95°C for 3 min, and 40 cycles of 95°C for 15 s and 60°C for 1 min.

### Western blot analysis

Cells from the six-well plates were detached and lysed with cell lysis buffer (50 mM Tris-HCl, 120 mM NaCl, 0.5% NP-40, 1 mM EDTA, 1 mM dithiothreitol (DTT), and 1 × protease inhibitor; Calbiochem). The samples were frozen and thawed and then centrifuged at 15,000 g at 4°C for 10 min. The supernatants were kept as whole cell samples. For cytoplasm/nuclear separations, the protocol reported previously was modified and used in this study [6,24], all cells from a T175 flask were lysed with 1 ml of hypotonic lysis buffer [10 mM Tris-HCl (pH 7.5), 10 mM NaCl, 3 mM MgCl_2_, 0.5% NP-40, and 1 × protease inhibitor] and centrifuged at 500*g* at 4°C for 5 min. This supernatant was kept as cytoplasmic extract. Nuclear pellets were washed with 1 mL hypotonic wash buffer [10 mM Tris-HCl (pH 7.5), 10 mM NaCl, 3 mM MgCl_2_, 2.5% NP-40, and 1 × protease inhibitor] with 5 min incubations on ice for three times.

To prepare nuclear extracts, nuclear pellets were resuspended in 0.5 mL of nuclear lysis buffer [50 mM Tris-HCl (pH 7.5), 10 mM EDTA, 1% sodium dodecyl sulfate (SDS), and 1 × protease inhibitor] and then sonicated (20% power, 20 s, 3 pulses). The samples were centrifuged at 15,000 g at 4°C for 10 min and the supernatant was kept as nuclear extracts (0.5 mL). Protein concentrations in whole cell, cytoplasmic, and nuclear samples were determined using the Pierce^TM^ BCA (Bicinchorinic acid) Protein Assay Kit (Thermo Fischer Scientific).

To measure protein expression, 20–30 μg of whole cell lysate and each fraction were analyzed by SDS-polyacrylamide gel electrophoresis (4%–20% TGX gels; Bio-Rad). Gels were run <50 V for 30 min and turned to 100 V for 2 h. After gel electrophoresis, proteins were transferred to nitrocellulose membrane (Amersham Protran; GE Healthcare) at 110 V for 100–120 min. After blocking the membrane with 5% nonfat dry milk/tris-buffered saline, 0.05% Tween20 detergent (TBST) at room temperature for 1 h, the membrane was incubated with primary antibodies at the following dilution ratio: anti-Dicer antibody (1:1,000, ab14601; Abcam), anti-TNRC6A (GW182) antibody (1:5,000, A302–329A; Bethyl Labs), anti-AGO2 antibody (1:2,000, 015-22411; Wako), antitubulin antibody (1:5,000, T5201; Sigma), anticalnexin antibody (1:1,500, 2433; Cell Signaling), antiataxin-3 (ATX-3) antibody (1:1,000, MAB5360; Millipore), anticyclooxygenase-2 (COX-2) antibody (1:1,000, 160112; CAYMAN CHEMICAL), anti-β-actin antibody (1:10,000, A5441; Sigma), and antihistone H3 (1:20,000, 2650S; Cell Signaling). Horseradish peroxidase (HRP)-conjugated antimouse immunoglobulin G (IgG) or rabbit IgG (1:1,000–10,000; Jackson ImmunoResearch) secondary antibody was used for visualizing proteins using SuperSignal Plus WestPico Chemiluminescent Substrate (Thermo Fischer Scientific). Protein bands were quantified using ImageJ software.

### Mass spectrometry

In solution, fractionation mass spectrometry (MS) [25,26] was used to estimate the number of protein copies per cell. Two flasks of cells were harvested and lysate in 600 μL solution containing 4% SDS, 100 mM Tris/HCl pH 7.6, and 0.1 M DTT. The lysate was incubated at 95°C for 5 min and then sonicated (20% power, 20 s, 2 pulses). The lysate was clarified by centrifugation at 16,000*g* at room temperature for 5 min. Then 200 μL of cell lysate was prepared using the filter-aided sample preparation method [25,27]. An Ultracel-10K (UFC501024; Millopore) filter was used for protein purification and trypsin digestion.

MS of the trypsinized peptides was performed by UT Southwestern proteomic core. An Ultimate 3000 RSLC nano-LC (Thermo Fischer Scientific) in-line connected to an Orbitrap Fusion Lumos (Thermo Fisher Scientific), for MS analysis. In brief, the sample was fractionated into 10 injections and peptides were loaded onto a reverse-phase column (Easy Spray column, either 75 μm × 50 cm or 75 μm × 75 cm, 2 μ beads). Peptides were loaded with solvent A (0.1% trifluoroacetic acid, 2% acetonitrile in water) and were separated with a linear gradient from 0% solvent A (2% acetonitrile, 0.1% formic acid in water) to 28% solvent B (0.1% formic acid, 80% acetonitrile, 10% trifluoroethanol, 10% water) at a flow rate of 250 nL/min for 60 min (for 50 cm column) or 90 min (for 75 cm column), followed by a wash reaching 99% solvent B for 5 min (50 cm column) or 25 min (75 cm column).

The MS was operated in data-dependent positive ionization mode, automatically switching between MS and MS/MS acquisition for the 10 most abundant peaks in a given MS spectrum. Full-scan MS spectra (m/z = 400–1,600) were acquired in the Orbitrap at a target value of 4E5 with maximum ion injection time of 50 ms, and a resolution of 120,000 at 200 m/z. The 10 most intense ions fulfilling a predefined criterion (AGC target 1E4 ions, maximum ion injection time of 100 ms, isolation window of 1.6 m/z, fixed first mass of 110 m/z, intensity threshold of 5E3, charge state = 2–7, peptide match preferred, exclude isotopes on, dynamic exclusion time of 25 s) were subjected to tandem MS scans in the ion trap using higher energy collisional dissociation (HCD) with a stepped collision energy of 33% ± 5%. Raw files were processed using MaxQuant and used the latest human database from Uniprot. We then used the label free quantization (LFQ, normalized intensity) data to calculate the number of protein copies per cell.

## Results

### Knockout cell lines

We selected HCT116 cells as the parental line for TNRC6 knockout variants because HCT116 is a diploid cell line well suited to knocking out multiple genes using CRISPR/Cas9-based methods. We obtained cell lines from GenScript that had the *TNRC6A* (Fig. 1A) and *TNRC6B* (Fig. 1B) genes knockout either individually or in combination.

Repeated attempts to obtain the TNRC6A/B/C triple knockout cell line were unsuccessful, suggesting that TNRC6 function is essential for survival of HCT116 cells. Supporting this suggestion, TNRC6A/B double knockout cells grow more slowly than single knockout or wild-type cells (Supplementary Fig. S2). To study the effect of removing TNRC6C protein, we transfected a fully complementary anti-TNRC6C siRNA, which efficiently inhibited *TNRC6C* gene expression (Supplementary Fig. S3). The TNRC6A/B knockout cells that also had a knockdown of TNRC6C grew approximately one-third more slowly than cells that were not treated with anti-TNRC6C duplex RNA.

Mammalian cells have three different paralogs of TNRC6A (six isoforms, four main isoforms; Fig. 1A), TNRC6B (three isoforms, one of which does not have an AGO-binding domain; Fig. 1B), and TNRC6C (two isoforms; Fig. 1C) [28]. TNRC6 must bind to AGO to participate in RNAi [11–13]. Therefore, to ensure a complete functional knockout of TNRC6A, a frameshift point insertion was introduced into the domain responsible for binding AGO protein at a site common to all four major isoforms. Sequencing confirmed the base insertion (Supplementary Fig. S1A).

For the TNRC6B knockout, a base pair insertion was inadequate and CRISPR-CAS9 was used to generate a 95,481 base-pair deletion spanning the AGO binding domain (Fig. 1B; Supplementary Fig. S1B, C). After generating both single mutations, we obtained double knockout cells with mutations in both TNRC6A and TNRC6B (Supplementary Fig. S1D, E). The double knockout cells contained the same TNRC6A frameshift mutation and the same TNRC6B deletion.

### MS to validate protein knockout

RNA expression is a complicated process that can produce variant RNAs and proteins. CRISPR produces mutations at the DNA and RNA level, but these mutations do not always guarantee that no functional protein will be produced. Assays that quantify protein expression are necessary.

The most direct method for analyzing protein expression is western blot analysis. We used an anti-TNRC6A antibody to demonstrate elimination of TNRC6A in TNRC6A knockout and TNRC6AB knockout cells (Supplementary Fig. S4). Unfortunately, we could not identify an anti-TNRC6B or anti-TNRC6C antibody adequate for conclusive western analysis. Therefore, we performed quantitative MS [27,29–31] to confirm the knockout of TNRC6B protein and to gain insights into how the knockout of TNRC6A or TNRC6B affects expression of other RNAi factors.

This quantitative MS approach uses the proteomic ruler method developed by Wisniewski and coworkers that permits label-free quantification for number of protein copies per cell [25]. The proteomic ruler method uses histones as a “ruler” to calculate the number of cell equivalents in a sample, because histones are proportional to the amount of DNA in a cell. This proportion is then used to calculate the number of protein copies based on the peptide intensity signal and molecular weight of the protein of interest.

These numerical estimates of number of protein copies are not exact but can be used to gauge how relative abundance differs as a function of the genetic background of a cell and to estimate the approximate number of protein molecules per cell. This capability is sufficient to achieve our goal—determining whether the target proteins were successfully knocked out. Additional replicates and more stringent control over experimental conditions would be necessary to confidently evaluate the importance of smaller differences in the detection of number of protein copies.

**FIG. 3.**
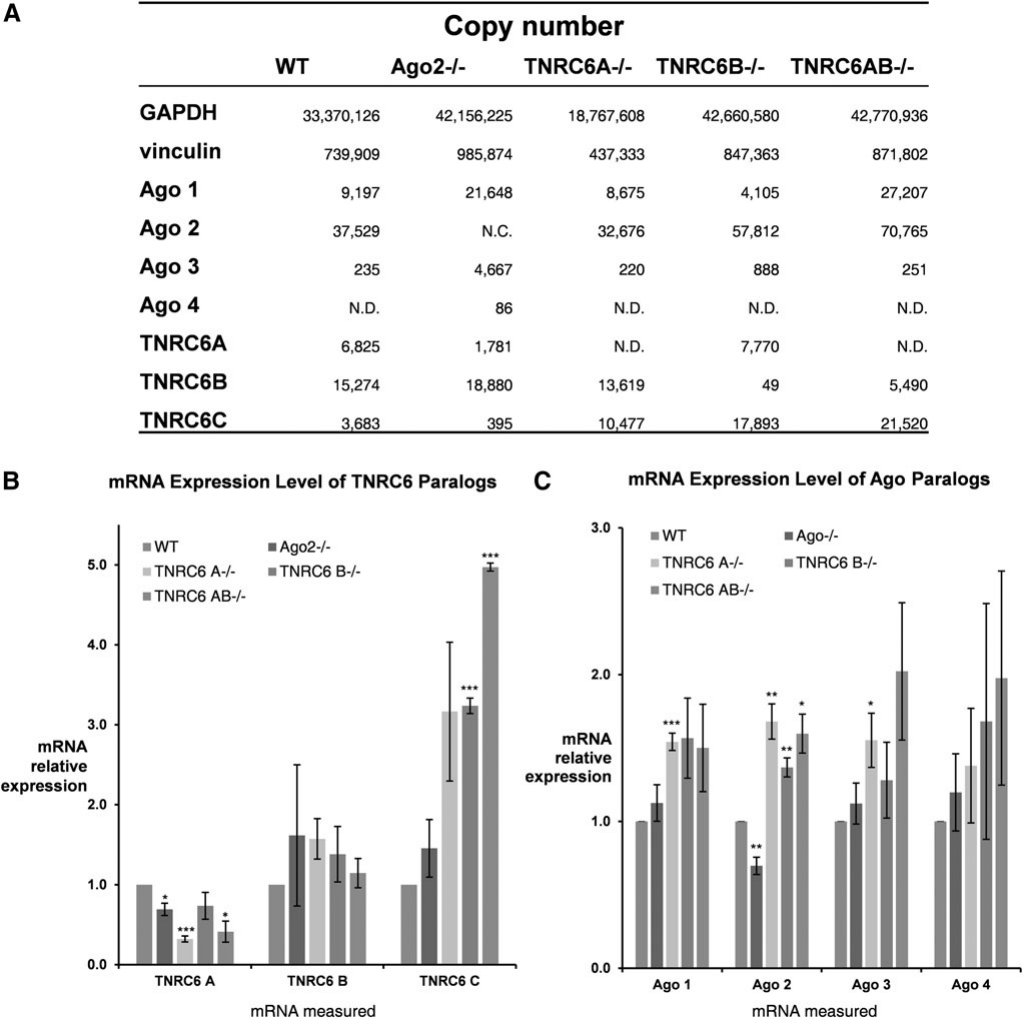
Protein and gene expression level of TNRC6 and AGO paralogs. **(A)** Table of protein copies for TNRC6 paralogs, AGO paralogs, and housekeeping genes. **(B)** mRNA levels for TNRC6 paralogs, *n* = 3. **(C)** mRNA level of AGO paralogs, *n* = 2. Error bars represent standard error of the mean. *P < 0.05; **P < 0.01; ***P < 0.001. NC, no confidence; ND, not detected; WT, wild-type. Data in graph is in order as follows: WT, Ago2^−/−^, TNRC6 A^−/−^, TNRC6 B^−/−^, TNRC6 AB^−/−^.

Our MS did not detect TNRC6A protein (Fig. 3A) in the TNRC6A knockout cells and the absence of TNRC6A was confirmed by western analysis (Supplementary Fig. S4). When single mutations were introduced into the *TNRC6B* gene, we did observe a substantial amount of TNRC6B protein (data not shown) because protein was produced by splice variants that did not contain the point mutation.

To generate a fully TNRC6B knockout cell line, CRISPR/CAS9 was used to generate a 95,481 base-pair deletion spanning the AGO binding domain of *TNRC6B*. In this TNRC6B knockout cell line, we detected one peptide of TNRC6B, but it belongs to the isoform 3, which does not contain the AGO binding domain, and is likely not relevant to investigation into the impact of TNRC6B on RNAi (Figs. 1B and 3A). Similarly, in the TNRC6A/B double knockout cell line, TNRC6B peptides belonging to isoform 3 were also detected and are also not likely to be relevant (Figs. 1B and 3A). These data confirm that the expression of functional TNRC6A and TNRC6B protein was successfully knocked out in HCT116 cells, both individually and as a double knockout.

### MS and qPCR reveal gene expression changes

Numerical data obtained by quantitative MS should be regarded as estimates for the number of protein molecules per cell. These estimates suggest trends when related cell lines are compared, for example, when a cell line with a TNRC6 knockout is compared with a wild-type cell line. Estimates for protein abundance changes can be independently checked by qPCR measurement of mRNA expression.

The data show that TNRC6A and TNRC6B are more highly expressed than TNRC6C in wild-type cells (Fig. 3A, B). TNRC6C expression increased dramatically when TNRC6A or TNRC6B was knocked out. The increase was as high as 10fold in the TNRC6AB double knockout cells (Fig. 3A). This increase was confirmed using qPCR (Fig. 3B).

These data suggest that the expression of TNRC6C is linked to levels of TNRC6A and TNRC6B. By contrast, in the TNRC6A^−/−^ cell line, the expression of TNRC6B mRNA did not significantly increase (Fig. 3B). Similarly, in the TNRC6B^−/−^ cell line, the expression of TNRC6A mRNA is similar to wild type. These results suggest that the expression of *TNRC6A* and *TNRC6B* genes is not linked.

We also used MS to evaluate the effect of the knockout of TNRC6 proteins on the expression of the TNRC6 paralogs and the four AGO variants found in human cells, AGO1–4. Because the TNRC6 paralogs can bind to the four AGO paralogs, we evaluated all four AGO variants through qPCR and MS.

AGO3 and AGO4 were present at low levels in both wild-type HCT116 and knockout cells and the knockout of TNRC6 proteins did not affect that low expression. Relative to AGO3 and AGO4, AGO1 and AGO2 are expressed at higher levels in wild-type cells at ∼9,000 and 37,000 copies per cell. MS suggested that expression of AGO1 and AGO2 was elevated in TNRC6AB knockout cells (Fig. 3A). qPCR also identified increases for both AGO1 and AGO2 expression in knockout cells (Fig. 3C). Conversely, the TNRC6 paralogs' mRNA expression is not altered significantly in the AGO2^−/−^ knockout cell lines.

### Effect of TNRC6 gene knockout on nuclear/cytoplasmic distribution of RNAi factors

RNAi can regulate gene expression in either the cytoplasm or nuclei of mammalian cells [24,32] and knocking out one or more critical RNAi factors may alter the localization of the remaining factors. To test this hypothesis, we used our knockout cell lines to examine the subcellular localization of RNAi factors in cytoplasmic and nuclear fractions.

To determine the purity of our nuclear/cytoplasmic extractions, β-tubulin (a cytoplasmic protein), calnexin [an endoplasmic reticulum (ER) marker], and histone H3 (a chromatin protein) were used as markers in western blot analysis. Calnexin was measured to ensure the ER was disassociated from the nucleus during the separation protocol because RNAi factors may associate with the ER. We were unable to remove the ER from HCT116 nuclei using published protocols [24] and it was necessary to develop a modified strategy (Materials and Methods section) to obtain pure nuclei.

**FIG. 4.**
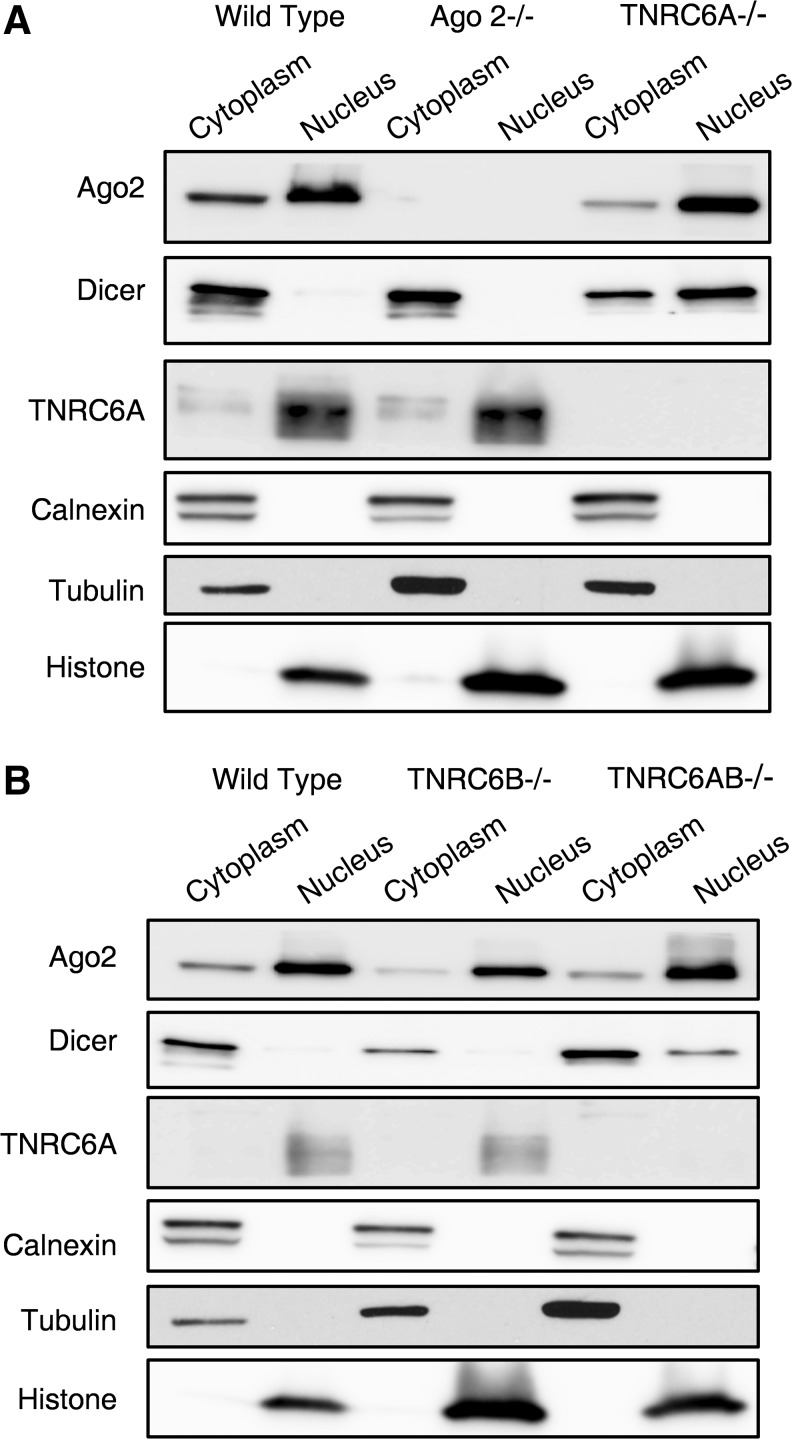
AGO2 and TNRC6 protein cellular distribution in different cell lines. **(A)**. Western blot of cytoplasmic and nuclear fractions from WT, AGO2^−/−^ and TNRC6A^−/−^ of calnexin, an ER protein, tubulin, a cytoplasm marker, and histone H3, a chromatin protein to show the purity of the extraction. **(B)** Western blot of cytoplasmic and nuclear fractions from WT, TNRC6B^−/−^, and TNRC6AB^−/−^ showing purity of the extraction. ER, endoplasmic reticulum.

In previously analyzed cell lines, we had observed that AGO2 was primarily localized in cell cytoplasm [24]. In HCT116 cells, most AGO2 is in cell nuclei (Fig. 4). Knocking out TNRC6A or TNRC6B did not substantially affect localization of AGO2—it remained in the nucleus. Western analysis also indicated that TNRC6A was primarily localized in cell nuclei. This nuclear localization was observed regardless of whether TNRC6B was present, suggesting that nuclear localization of TNRC6A is independent of TNRC6B.

In contrast to the predominantly nuclear localization of AGO2 and TNRC6A, Dicer was almost entirely in the cytoplasm of wild-type cells (Fig. 4). This is consistent with Dicer's known role in RNA processing. However, knocking out either TNRC6A or TNRC6AB, and knockdown TNRC6C in the TNRC6AB^−/−^ cells, but not TNRC6B alone, caused an increase in the nuclear localization of Dicer (Fig. 4; Supplementary Fig. S5). These data suggest that TNRC6A may play a role in subcellular localization of Dicer and that there are differences between the biological impact of TNRC6A and TNRC6B.

### TNRC6 paralogs are not needed for gene silencing by fully complementary RNA duplexes

Fully complementary duplex RNAs are commonly used to silence the expression of target RNA transcripts. To test the effect of our gene knockouts on RNA silencing, we selected two well-characterized examples of duplex siRNA silencing: an mRNA encoding ATX-3 and the nuclear noncoding RNA MALAT-1. ATX-3 and MALAT-1 were chosen for analysis because they (1) represent both cytoplasmic and nuclear targets, (2) are ubiquitously expressed, and (3) do not play critical roles in the proliferation of HCT116 cells. We had previously reported that knockdown of TNRC6A/B/C did not reverse gene silencing of these targets, but one criticism of these results was that the substantial amount of TNRC6 protein remaining after triple knockdown with duplex RNAs was sufficient to support RNAi. Protein knockouts were needed to achieve more definitive conclusions.

**FIG. 5.**
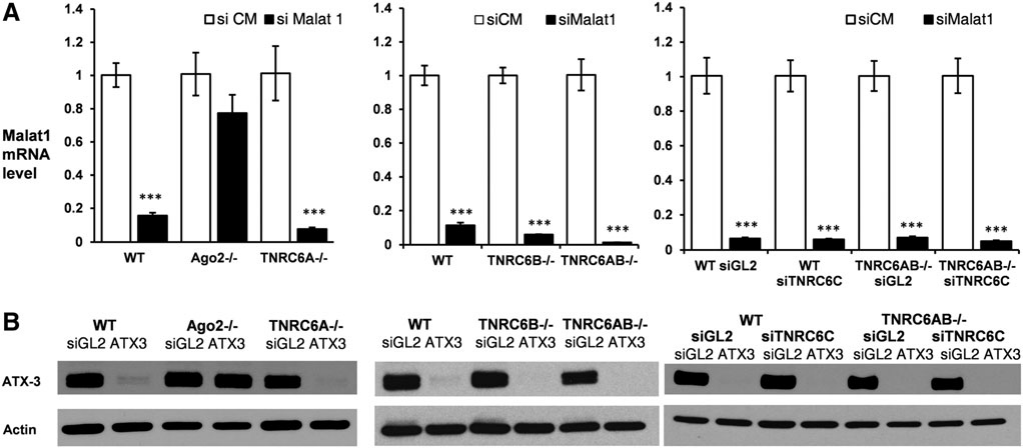
Assay of siRNA pathways in TNRC6 knockout cell lines. **(A)** siRNA-mediated inhibition of nuclear noncoding RNA MALAT1 expression, *n* = 3. Each measurement using siMALAT1 is normalized to treatment with the corresponding siCM control RNA. **(B)** Western blot analysis of siRNA-mediated inhibition of ATX3 protein expression. Error bars represent standard deviation, ***P < 0.001. siCM and siGL2 are noncomplementary duplex RNAs.

We also examined the effects of knocking out AGO2. AGO2 is the catalytic engine of RNAi [33] and would be expected to play a critical role in gene silencing. AGO2 binds the guide strand of duplex RNA and enzymatically cleaves the RNA target strand after recognition when there is full complementarity between the central region of guide strand and target RNA (Fig. 2A, B). As expected, knocking out AGO2 reversed gene silencing of both MALAT-1 (Fig. 5A) and ATX-3 (Fig. 5B).

By contrast, knocking out TNRC6A, TNRC6B individually, or TNRC6A and TNRC6B in combination had no effect on gene silencing (Fig. 5). Addition of an siRNA that silences 94% of TNRC6C expression (Supplementary Fig. S3B) to TNRC6A/B knockout cells also had no effect on gene silencing of either target RNAs. These data are consistent with the conclusion that TNRC6 proteins are not necessary for the action of fully complementary duplex RNAs, regardless of whether the target is predominantly nuclear or cytoplasmic.

### Requirement for TNRC6 paralogs during translational silencing by miRNAs

**FIG. 6.**
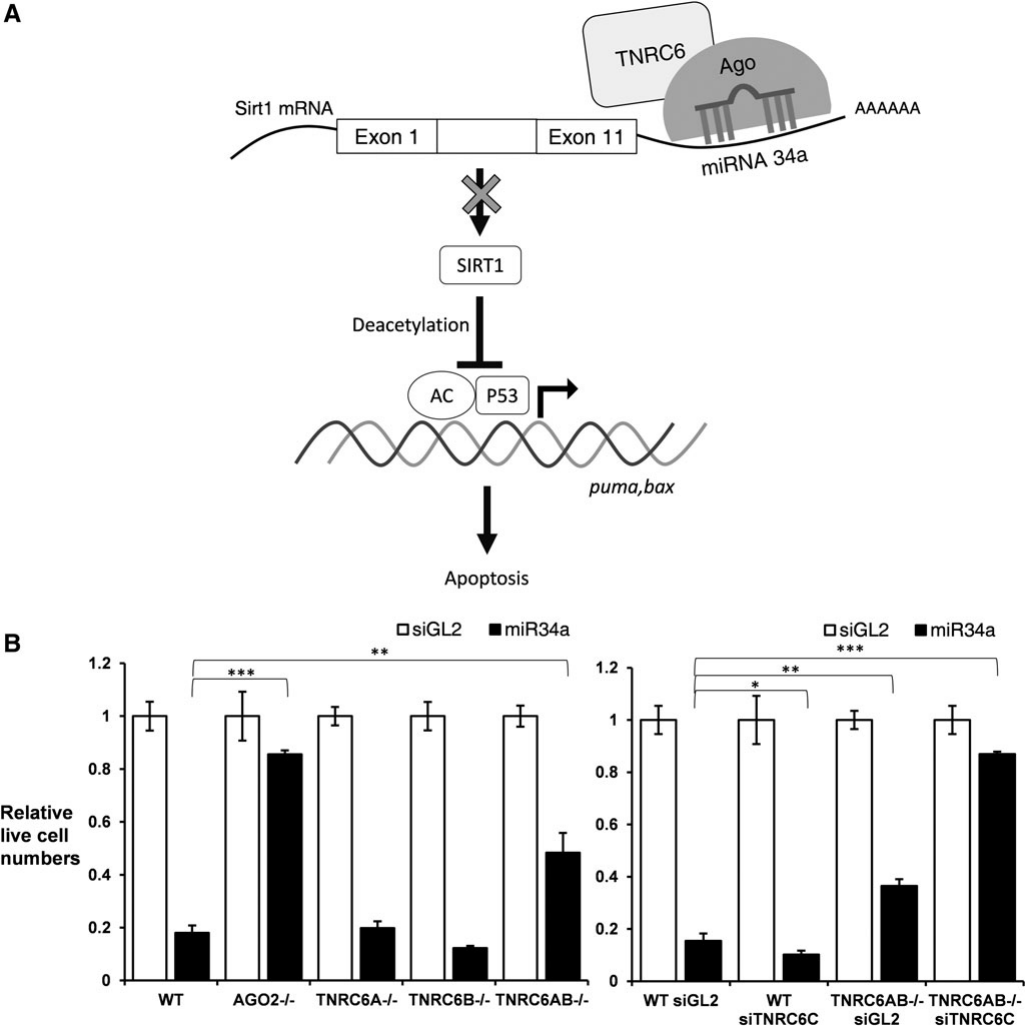
Assay of miRNA pathway in TNRC6 knockout cell lines. **(A)** Schematic of miR34a pathway. **(B)** Quantification of live cells after treating cells with an miR34, *n* = 3. Each treatment with the miR-34a mimic is normalized to a parallel treatment with a non complementary RNA duplex. Error bars represent standard deviation, *P < 0.05; **P < 0.01; ***P < 0.001. siGL2 is a non complementary control duplex RNA.

miR-34a is partially complementary to the sirtuin 1 (SIRT1) 3′-untranslated region and inhibition of SIRT1 can induce cell apoptosis (Fig. 6A) [34–36]. SIRT1 inhibits p53-dependent apoptosis by deacetylating major acetylation sites on p53. When p53 is acetylated, it activates genes involved in apoptosis, such as puma and bax. miR-34a is also a direct gene target of p53, so its introduction into the cell creates a positive feedback loop where miR-34a activates p53, which then activates further miR-34a transcription. We chose miR-34a to examine the effects of knocking out TNRC6 paralogs because it is a well-characterized miRNA that has a robust impact within cells, resulting in cell death that can be monitored quantitatively. To measure the relative amount of cell death, we counted the number of live cells 72 h after transfection.

We observed that addition of synthetic miR34a reduced number of live cells dramatically in wild-type HCT116 cells but not in the AGO2 knockout cells, consistent with the involvement of AGO2 in miRNA action. Knocking down TNRC6C or knocking out TNRC6A or TNRC6B individually had no effect on the activity of miR-34a. Knocking out both TNRC6A and TNRCB partially reversed miR-34a action. When knockout of TNRC6A and TNRC6B is combined with siRNA-mediated TNRC6C silencing, miR-34a activity was almost entirely reversed (Fig. 6B).

These data suggest that the TNRC6 paralogs have redundant functions during gene silencing by miRNAs. The ability of TNRC6C alone to partially support activity even when both TNRC6A and TNRC6B are knocked out is consistent with our observation that TNRC6C expression is increased when TNRC6A and TNRC6B expression is abolished (Fig. 3).

### Requirement for TNRC6 paralogs during RNA-mediated transcriptional activation

We have previously observed that the expression of *COX-2* gene transcription can be activated by small duplex RNAs that are target to the *COX-2* promoter [37]. This mechanism of RNA-mediated transcriptional activation involves the guide strand RNA binding to a noncoding RNA that overlaps the TATA box at the *COX-2* promoter (Fig. 2C). Binding induces histone modifications, and, in A549 lung cancer cells, activation can be up to 30-fold.

**FIG. 7.**
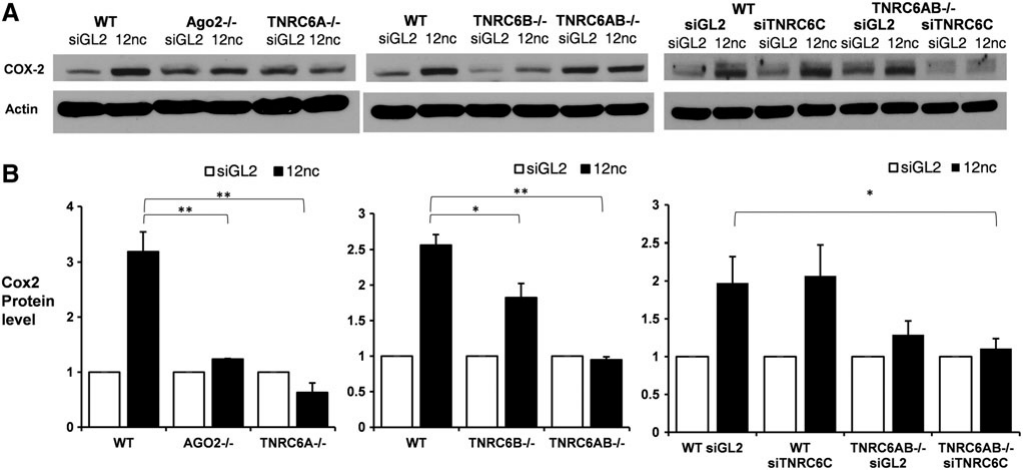
Effect of knocking out TNRC6 paralogs on transcriptional activation of COX-2 protein expression by a promoter-targeted duplex RNA. **(A)** Western blot analysis of activation of COX-2 protein expression, *n* = 2. **(B)** Quantification of data in (A). Error bars represent standard deviation, *P < 0.05; **P < 0.01. siGL2 is a non complementary control duplex RNA.

HCT116 cells are not a characterized model for COX-2 expression. However, we did establish that threefold enhanced expression could be achieved upon addition of promoter-targeted duplex RNA (Fig. 7) but not RNA siGL2, a noncomplementary control. Several other control duplexes had been used previously to confirm selectivity and establish mechanism [37].

We observed that the basal expression of COX-2 varied between the different knockout cell lines (Supplementary Fig. S6). Therefore, RNA-mediated activation was normalized to the basal expression level, as seen in the siGL2 band, for each individual cell line. Knocking out AGO2 reversed gene activation (Fig. 7), consistent with the necessity for AGO2 to assist binding of the target transcription by the small promoter-targeted RNA [22].

We observed no RNA-mediated activation when TNRC6A was knocked out in any context: alone, in combination with TNRC6B, or TNRC6AB^−/−^ with TNRC6C knockdown (Fig. 7). Activation was observed when TRNC6B was knocked out and when TNRC6C was knocked down. These results suggest that both TNRC6A and AGO2 are essential for RNA-mediated gene activation at the *COX-2* promoter. TNRC6B and TNRC6C expression do not appear to be essential.

## Discussion

### Using CRISPR in tandem with quantitative MS

We had previously examined the roles of TNRC6 proteins using siRNA-mediated knockdown of gene expression [22]. The weakness of these experiments was that 10%–20% of cellular TNRC6A or TNRC6B expression remained after knockdown. To obtain more definitive results, we used CRISPR to knock out TNRC6A and TNRC6B, individually and in combination. The triple knockout TNRC6A/B/C could not be obtained after repeated attempts, suggesting complete elimination of all TNRC6 expression is detrimental to cell growth.

We used quantitative MS [25–27,29,30] to evaluate protein expression to (1) validate that the gene knockouts at the level of protein expression and (2) to obtain information on how gene knockouts affect the expression of other proteins. We learned some valuable general lessons about the powerful combination of CRISPR and MS. MS proved to be the most general method for detecting protein expression and confirming a comprehensive knockout of isoform expression. It allowed us to estimate the number of protein copies per cell to afford more insight into cells.

The initial CRISPR knockout strategy for TNRC6B involved making point mutations or small deletions in the *TNRC6B* gene. The *TNRC6A* and *TNRC6B* genes were sequenced to confirm the CRISPR mutations, but sequencing does not ensure that alternatively spliced but potentially functional TNRC6A and TNRC6B proteins are not expressed in the cells. Quantitative MS revealed that the knockout was incomplete because TNRC6B isoforms that were alternatively spliced to exclude the mutated exon were still produced.

Because of the flexibility of the alternative splicing in the mammalian genome, indels may not stop protein expression. The detection of the planned mutation by sequence or the detection by qPCR of what had been assumed to be a primary splice variant does not guarantee that no relevant protein variant is being expressed. After realizing this shortcoming for TNRC6B, a second CRISPR knockout strategy that deleted a much larger section of the gene was successful.

We also gained an appreciation for the strengths and limitations for quantitative MS. This powerful technique is not well suited for generating exact values for the number proteins per cell. We observed substantial variation from one replicate to the next. Although the exact number must be treated with caution, the amount of expression for proteins relative to one another was informative and does suggest trends that can be further validated by qPCR measurement of mRNA expression. The technique also appears to be reproducible between laboratories, as our results in HCT116 are similar to those earlier obtained in HeLa cells and human melanoma cells [38,39].

### Effect of TNRC6 knockout on protein expression and localization

In wild-type cells, TNRC6C protein expression is lower than that of TNRC6A or TNRC6B (Fig. 3). When TNRC6A and TNRC6B are knocked out alone or in tandem, we observe an increase in TNRC6C expression. These data suggest that maintaining TNRC6 expression is important for cell growth and survival (Fig. 6B; Supplementary Fig. S2). We hypothesize that loss of TNRC6A and TNRC6B activates TNRC6C expression. Upregulated TNRC6C protein can offset the deficits in TNRC6A and TNRC6B protein expression. By contrast, knocking out TNRC6A does not appear to greatly affect expression of TNRC6B and *vice versa*.

Using stringent protocols for obtaining pure cell nuclei, we have previously observed that RNAi factors AGO2, TNRC6A, and Dicer are localized in both the cytoplasm and the nucleus in multiple cell lines [4,24]. One important technical lesson from this study is that our previously published protocol for isolation of pure nuclei is not sufficient for every cell line. We found that it was much more difficult to remove contaminating ER from the nuclei of HCT116 cells, emphasizing that the success of nuclei purification cannot be assumed, and that optimization may be necessary.

AGO2, TNRC6, Dicer, and other RNAi factors can be found in the cell nuclei [5,6,24,32]. Nuclear localization sequences and nuclear export signals have been reported in TNRC6 paralogs, indicating that the paralogs might affect cellular localization of AGO or the other proteins [6,40,41]. TNRC6 proteins are transported into nucleus by importin family and out of nucleus mediated by exportin 1 [41,42].

Our purification of HCT116 nuclei confirmed the presence of AGO2 and TNRC6A in cell nuclei. Indeed, these two proteins were more abundant in nuclei than in cytoplasm. Dicer was not observed in wild-type HCT116 nuclei but was observed in nuclei when TNRC6A and TNRC6B were knocked out. The distribution of AGO2 was not affected by knockout of TNRC6A or TNRC6B, alone or in combination, suggesting that these proteins are not involved with nuclear uptake. These results contrast with previously published results, suggesting that TNRC6A plays an important role in the nuclear entry of AGO2 [40]. It is possible that nuclear import of AGO2 may differ depending on cell line and growth conditions.

### Effect of knocking out TNRC6 on different RNAi mechanisms for controlling gene expression

The duplex RNAs used in current therapeutic development programs and in the one approved duplex RNA drug are fully complementary to their target sequences [43]. In this study, we observe that the action of fully complementary RNAs is independent of TNRC6A, TNRC6B, and TNRC6C alone or in combination. Our data confirm that AGO2 is necessary for gene silencing by these fully complementary RNAs and suggest that TNRC6 proteins and their ability to recruit additional factors through their scaffolding ability are not necessary. By contrast, gene silencing by miR-34A required expression of at least some TNRC6 protein, consistent with the need for TNRC6 protein as scaffold for binding the CCR4-NOT complex to achieve translation silencing by miRNA [22].

Our data suggest that TNRC6A paralog is required for RNA-mediated transcriptional activation of COX-2. This result is supported by TNRC6's function as a central organizer and scaffolding protein [4]. The necessity for TNRC6A contrasts with our previously published results [22] reporting that the knockdown of TNRC6A had no effect on RNA-mediated gene activation of COX-2. The discrepancy between the results from knockdown and knockout experiments underlines the value of pursuing experiments in knockout cell lines in addition to the more convenient experiments using gene knockdown using siRNAs.

### Conclusions

Relative to gene knockdown, CRISPR gene knockouts provide a more definitive strategy for understanding gene function. The combination of CRISPR with quantitative MS can validate gene knockouts and provide insights into broader protein expression trends. AGO2 is critical for every RNAi mechanism, whereas the TNRC6 paralogs are necessary for the action of miRNAs but not for fully complementary RNAs similar in design to those in the clinic. For gene activation, TNRC6A, but not TNRC6B or TNRC6C, is necessary, suggesting some differences in the ability of the paralogs to affect gene expression.

## Supplementary Material

Supplemental data

Supplemental data

Supplemental data

Supplemental data

Supplemental data

Supplemental data
