# Supplementary material for: Expression of TNRC6 (GW182) Proteins Is Not Necessary for Gene Silencing by Fully Complementary RNA Duplexes
Source: Nucleic Acid Ther. 2019 Dec 2;29(6):323–34. doi: 10.1089/nat.2019.0815 (PMC6885777; doi:10.1089/nat.2019.0815)
Supplement: Supplemental data [file Supp_Table1.pdf]

Table 1. siRNA sequence.

siRNA name		siRNA sequence
siGL2	AS	UCGAAGUAUUCCGCGUACGdTdT
	SS	CGUACGCGGAAUACUUCGAdTdT
siCM	AS	GAGAAAGAGAAAGGAAAGdTdT
	SS	CUUUCCUUUUCUCUUUCUCdTdT
siAGO2 - 1	AS	UUCAGAUGGACUUCCGUGCUU
	SS	GCACGGAAGUCCAUCUGAAUU
siAGO2 - 2	AS	UAAUACAUCUUUGUCCUGCUU
	SS	GCAGGACAAAGAUGUAUUAUU
siAGO2 - 3	AS	UAUUUAUCACCACAGACCCUU
	SS	GGGUCUGUGGUGAUAAAUAUU
siAGO2 - 4	AS	UGACAUUGGGUUCUCAUACUU
	SS	GUAUGAGAACCCAAUGUCAUU
siTNRC6C - 1	AS	AAGUGGACGUUUGUGGUUCdTdT
	SS	GAACCACAAACGUCCACUU
siTNRC6C - 2	AS	UACUGAUGUCAACCUGGAAGUGUAGAA*
	SS	CUACACUUCCAGGUUGACAUCAGA
siTNRC6C - 3	AS	UGACAUUCAUGUUUGGGUUCAGUCCAG*
	SS	GGACUGAACCCAAACAUGAAUGUCA
siATX3	AS	GUAACUCCUCCUUCUGCCAdTdT
	SS	UGGCAGAAGGAGGAGUUAG
siMALAT1	AS	UUACCAACCACUCGCUUUCdTdT
	SS	GAAAGCGAGUGGUUGGUAAdTdT
12nc	AS	GACAAUUGCACGCUAACCGdTdT
	SS	CGGUUAGCGUGCAAUUGUC
miR34a		UGGCAGUGUCUUAGCUGGUUGU

All the sequences are listed 5’ to 3’. Underlined bases are mismatched.
Sequences labeled with * are from IDT.
