# Supplementary material for: Expression of TNRC6 (GW182) Proteins Is Not Necessary for Gene Silencing by Fully Complementary RNA Duplexes
Source: Nucleic Acid Ther. 2019 Dec 2;29(6):323–34. doi: 10.1089/nat.2019.0815 (PMC6885777; doi:10.1089/nat.2019.0815)
Supplement: Supplemental data [file Supp_Table2.pdf]

Table 2. q-PCR primers sequence

q-PCR primers **name**	q-PCR primers **sequence**
HPRT-F	AGTTCTGTGGCCATCTGCTTAGTAG
HPRT-R	AAACAACAATCCGCCCAAAGG
RPL19-F	AGCCTGTGACGGTCCATTCC
RPL19-R	CGGCGCAAAATCCTCATTCT
SNRNP-F	CGAGAAGTGGGACATCATCAC
SNRNP-R	ACCTCTGTCATCGTGGAGAA
Ago1-F	TGTTACCTCACTGGATAGAAGAAAG
Ago1-R	AGGAAACAAGGCATCCTACAC
Ago2-F	GCTACACTCAGACCAACAGATG
Ago2-R	GACGGAAGGCATTCTGGAAA
Ago3-F	CTCTGAGAGGAAGTACTGAAAGATG
Ago3-R	CACACAGTGTTGGTTTCTGTATG
Ago4-F	CTACCAGCAGCTCGGAATAG
Ago4-R	TCAGTCCAGTGCATCAGAAA
TNRC6A-F	AGCAAGCACAGGTACATCAG
TNRC6A-R	CAGTTGTGGCTGGAGTAGAAG
TNRC6B-F	TGACCCTGAATCTGACCCCTATG
TNRC6B-R	TGCTGAAGTGCTATGAACGTTGG
TNRC6C-F	CTGGAGGTCTAAGCATTGGGC
TNRC6C-R	TCAGGGTCATTCTCAGGGTCAA
Malat1-F	CGGGTGTTGTAGGTTTCTCTT
Malat1-R	CCCACAAACTTGCCATCTACTA
