# Supplementary material for: Expression of TNRC6 (GW182) Proteins Is Not Necessary for Gene Silencing by Fully Complementary RNA Duplexes
Source: Nucleic Acid Ther. 2019 Dec 2;29(6):323–34. doi: 10.1089/nat.2019.0815 (PMC6885777; doi:10.1089/nat.2019.0815)
Supplement: Supplemental data [file Supp_Fig1.pdf]

Supplementary Data

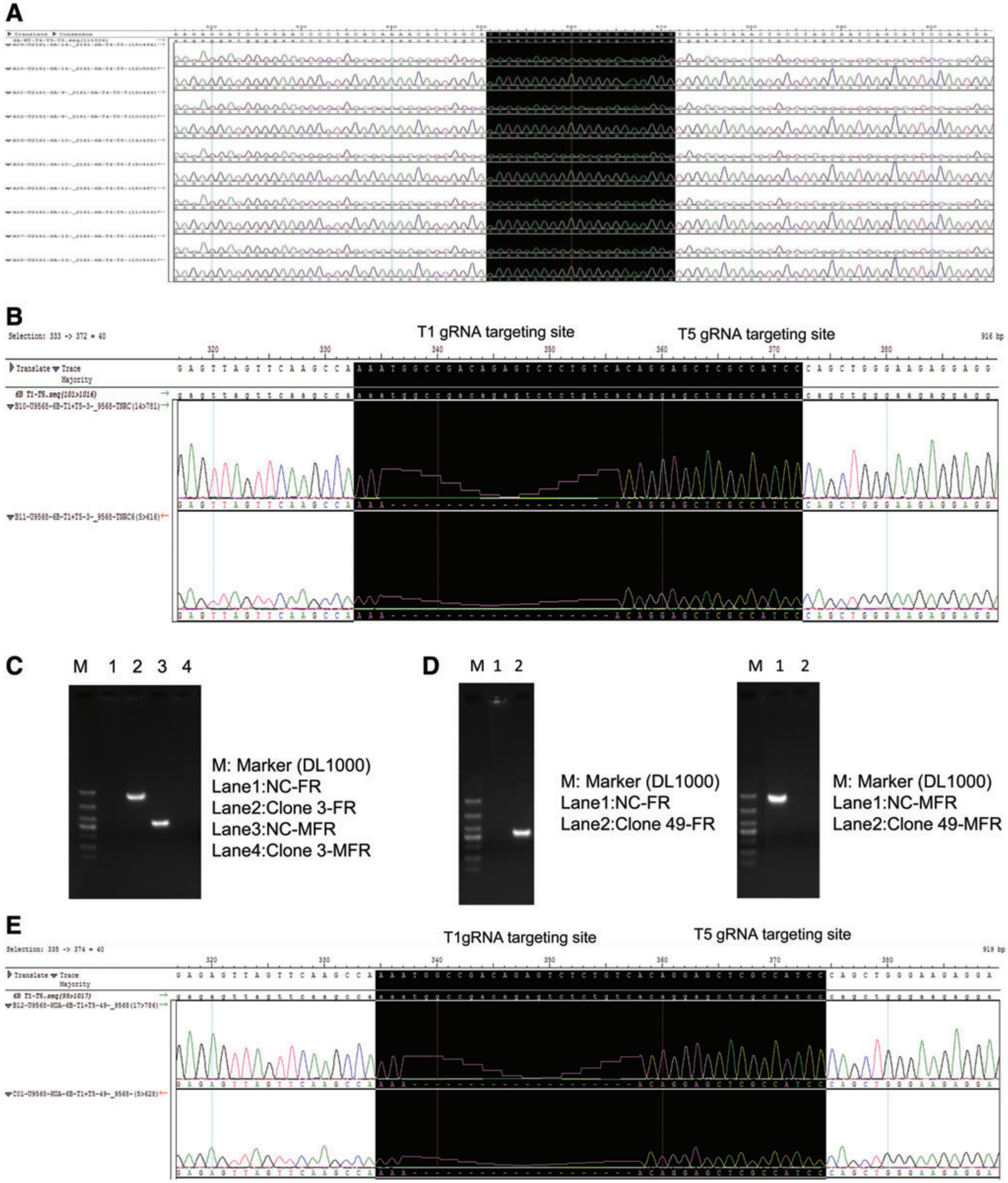

SUPPLEMENTARY FIG. S1. Confirmation of knockout cell lines. **(A)** Sequence data of TNRC6A$^{-/-}$ knockout cells (1 bp insertion). **(B)** Sequence data of TNRC6B$^{-/-}$ knockout cells (95,481 bp deletion). **(C)** DNA gel data of TNRC6B$^{-/-}$ clone 3 (NC-WT cell, FR primers outside the deletion part, MFR primers inside the deletion part). **(D)** DNA gel data of TNRC6AB$^{-/-}$ clone 49 (NC-WT cell, FR outside the deletion part, MFR inside the deletion part). **(E)** Sequence data of TNRC6AB$^{-/-}$ knockout cells (95,481 bp deletion on TNRC6B gene). bp, base pair.
