# Supplementary material for: Expression of TNRC6 (GW182) Proteins Is Not Necessary for Gene Silencing by Fully Complementary RNA Duplexes
Source: Nucleic Acid Ther. 2019 Dec 2;29(6):323–34. doi: 10.1089/nat.2019.0815 (PMC6885777; doi:10.1089/nat.2019.0815)
Supplement: Supplemental data [file Supp_Table3.pdf]

Table 3. sgRNAs and PCR primers sequence.

**sgRNA name**	**sgRNA sequence**
TNRC6A-1 *	CTAATTTACCCAGCGTTGAG TGG
TNRC6A-2	AGTGGAAATGGCGCAAATTC TGG
TNRC6A-3	AACTCCCAGAGTACATCATG GGG
TNRC6B-1 *	AGAGACTCTGTCGGCCATTT TGG
TNRC6B-2	TTCGGGTAGGTTTGTCAATC AGG
TNRC6B-3	AAGCACTGCTGCACTTTATG AGG
TNRC6B-4	AATCTATGCAAGACGGCTGG GGG
TNRC6B-5 *	GGATGGCGAGCTCCTGTGAC TGG
TNRC6B-6	GACTCGCTGAGCAACCTCCT GGG
PCR primers **name**	PCR primers **sequence**
TNRC6B-T1-F	AAGCGAGCCAGGAGAAGAT
TNRC6B-T5-R	ACGAAGGGTAAAAGGGAAGTA
TNRC6B-T5-F	TCCCAACCCCTCTCTTTA
TNRC6B-T1-R	AATGGCTTTCCTGGACTTTA

Sequences labeled with * are used for knocking out TNRC6 gene.
