# Supplementary material for: Expression of TNRC6 (GW182) Proteins Is Not Necessary for Gene Silencing by Fully Complementary RNA Duplexes
Source: Nucleic Acid Ther. 2019 Dec 2;29(6):323–34. doi: 10.1089/nat.2019.0815 (PMC6885777; doi:10.1089/nat.2019.0815)
Supplement: Supplemental data [file Supp_Fig2-4.pdf]

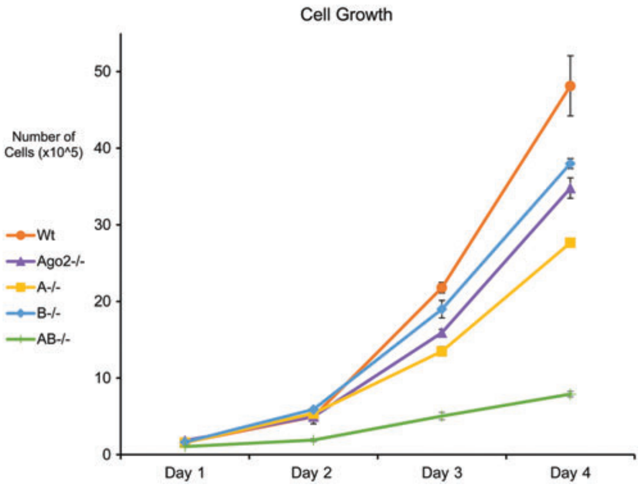

SUPPLEMENTARY FIG. S2. Cell growth rate, $n = 3$.

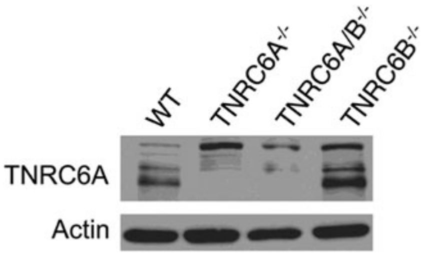

SUPPLEMENTARY FIG. S4. TNRC6A protein expression level in different cell lines. The upper band seen in all cell lines is a nonspecific band.

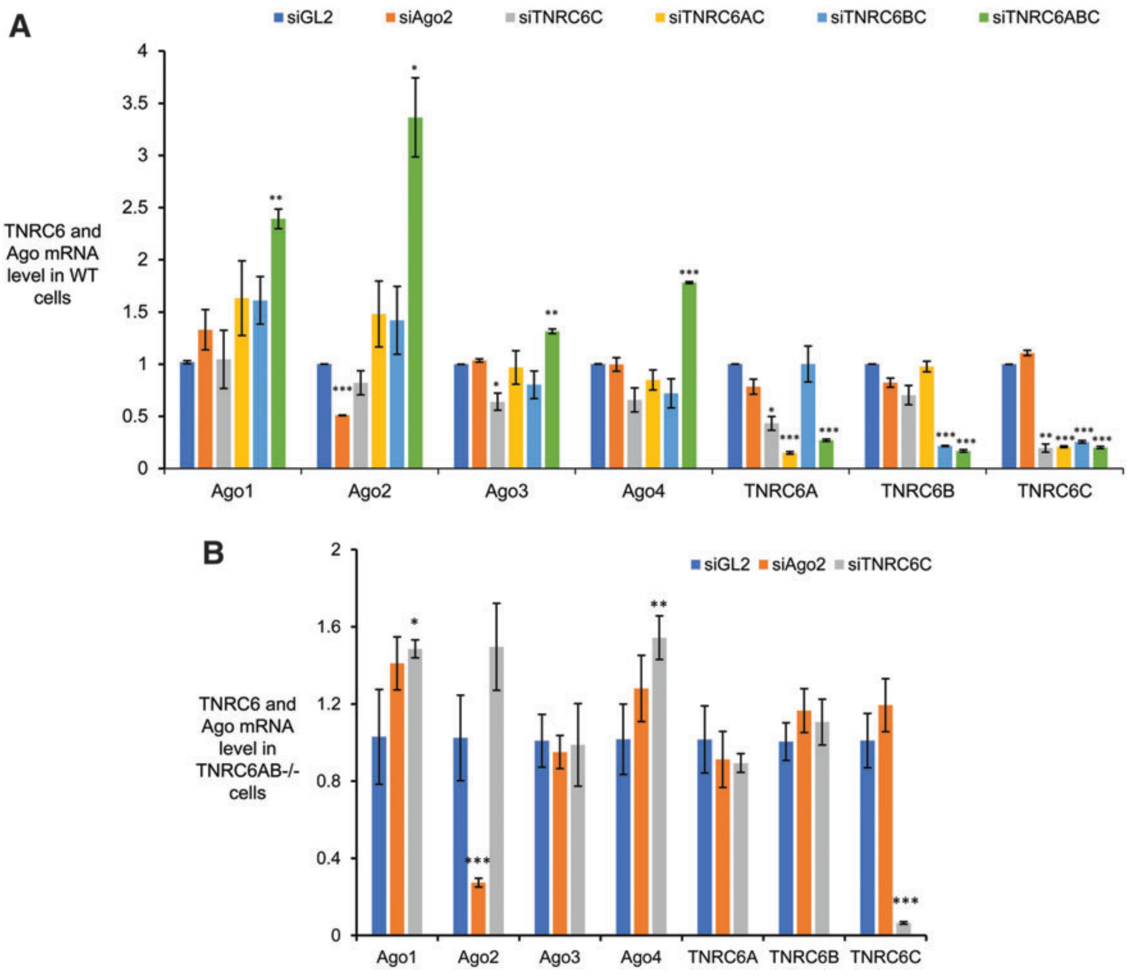

SUPPLEMENTARY FIG. S3. Knocking down the TNRC6C in WT and TNRC6AB$^{-/-}$ cell lines. **(A)** Gene expression level changes in WT cells, $n = 2$, error bars are standard deviation. **(B)** Gene expression level changes in TNRC6AB$^{-/-}$ cells, $n = 2$, error bars are standard deviation. *Means $p < 0.05$, **means $p < 0.01$, ***means $p < 0.001$. WT, wild type.
