# Supplementary material for: Expression of TNRC6 (GW182) Proteins Is Not Necessary for Gene Silencing by Fully Complementary RNA Duplexes
Source: Nucleic Acid Ther. 2019 Dec 2;29(6):323–34. doi: 10.1089/nat.2019.0815 (PMC6885777; doi:10.1089/nat.2019.0815)
Supplement: Supplemental data [file Supp_Fig5-6.pdf]

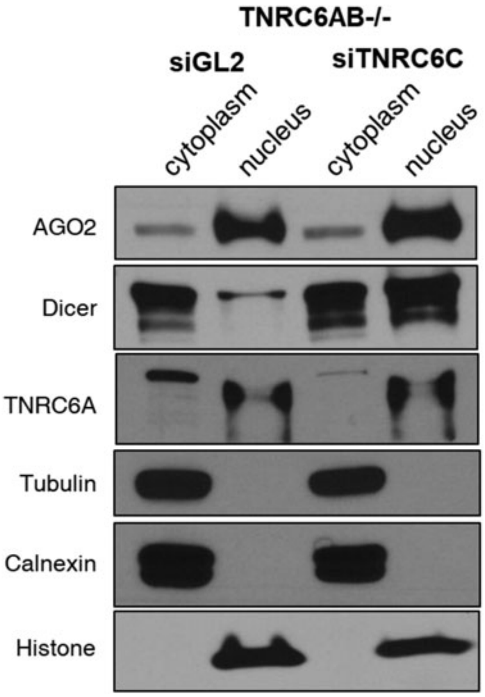

SUPPLEMENTARY FIG. S5. Cytoplasm and nucleus extraction in TNRC6AB double-knockout cells transfected with siTNRC6C. Proteins evaluated were calnexin, an ER protein, tubulin, a cytoplasm marker, and histone H3, a chromatin protein to show the purity of the extraction. ER, endoplasmic reticulum.

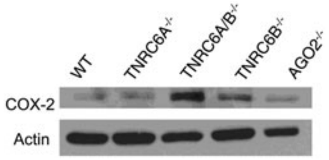

SUPPLEMENTARY FIG. S6. The basal level of COX-2 protein in different cell lines.

Supplementary Table S1. Small Interfering RNA Sequence

Supplementary Table S2. Quantitative PCR Primers Sequence

Supplementary Table S3. Single Guide RNAs and PCR Primers Sequence
